# A Systems Analysis of the Relationships Between Anemia and Ischemic Stroke Rehabilitation Based on RNA-Seq Data

**DOI:** 10.3389/fgene.2019.00456

**Published:** 2019-05-24

**Authors:** Yingying Wang, Xingxian Huang, Jianfeng Liu, Xuefei Zhao, Haibo Yu, Yunpeng Cai

**Affiliations:** ^1^Research Center for Biomedical Information Technology, Shenzhen Institutes of Advanced Technologies, Chinese Academy of Sciences, Shenzhen, China; ^2^Shenzhen Traditional Chinese Medicine Hospital, Shenzhen, China; ^3^Department of Neurology, The First Affiliated Hospital of Harbin Medical University, Harbin, China; ^4^Institute of Harbin Hematology & Oncology, The First Hospital of Harbin, Harbin, China

**Keywords:** hemoglobin, ischemic stroke, bioinformatics, anemia, predictive

## Abstract

Ischemic stroke (IS) is one of the main causes of morbidity and disability worldwide due to its complex mechanism. Anemia was characterized as a risk factor of IS because the direct connection between central nervous system, blood supply, and tissue oxygen delivery. As the key oxygen-carrying molecule in the blood, hemoglobin (Hb) may be decisive in the destiny of penumbral area or influence the brain recovery and neurologic function, which could finally affect the outcome of IS. However, more detailed information on the expression levels of Hb related genes were still lacking possibly because the concentration of Hb was determined by the genes’ expression several hours ago, which may make the research more difficult to perform. This time gap between gene expressions and protein concentration could make these genes predictive bio-markers for IS outcome. In this study, we choose 28 IS patients, of which 12 were suffering from anemia. Statistical analysis results showed that the outcomes of the patients were different when dividing them into two groups characterized by Hb concentration. 2 sex and age matched patients were first chosen to perform RNA-seq analysis on, on two occasions at two different time points, after which the Hb counts were tested at least 24 h after sequencing. Results showed that the outcome of anemia patients was poor compared with non-anemia patients. Two other patients were then chosen for analysis which excluded the coincidence of other factors. The results showed that the low value of Hb under 13 g/dL in men were closely related to the poor outcome of IS patients. Differently expressed Hb related genes were tested and six genes were shown to be positively correlated with the recovery degree of IS patients: ELANE, FGF23, HBB, PIEZO1, RASA4, and PRTN3. Gene CPM was shown to be negatively correlated with clinical outcomes. All of the seven genes were validated to be related to strokes using real-time PCR or literature searches. Taken together, these genes could be considered as new predictors for the recovery of IS patients.

## Introduction

Ischemic stroke (IS) is one of the main causes of morbidity and disability worldwide due to its complex mechanism. Many bio-markers and risk factors had been successfully identified using different methods (Jiang et al., 2018a,b, 2019). Similarly, alongside smoking, diabetes mellitus, hypertension, and hypercholesterolemia, anemia was characterized as ‘the fifth cardiovascular risk factor’ (Kaiafa et al., 2015). For example, the mortality rate was shown to be significantly higher in atherosclerosis-related IS patients suffering from anemia when admitted (Huang et al., 2009). The results of ASTRAL (Acute Stroke Registry and Analysis of Lausanne) showed that anemia on admission could predict both short-term and long-term outcomes in patients with IS and the risk of recurrent stroke was higher in anemia patients (Milionis et al., 2015).

The relationships between IS and anemia may be explained partly by the direct connection between central nervous system (CNS), blood supply, and tissue oxygen delivery (Dubyk et al., 2012). A sudden interruption of the oxygen supply to brain tissue was a crucial step in the pathophysiology of IS. The regaining of oxygen supply to ensure timely reperfusion or collateral perfusion would then determine the conditions of brain tissues (Kellert et al., 2011). As the key oxygen-carrying molecule in the blood, hemoglobin (Hb) may be decisive for the destiny of penumbral area or influence the brain recovery and neurologic function, which could finally affect the outcome of IS (Kimberly et al., 2013; Park et al., 2013). It had been shown that poor outcome and mortality after IS were strongly associated with low and further decreasing hemoglobin (Hb) and hematocrit (Hct) levels (Kellert et al., 2011). Another study found that the poor outcome of acute IS was related to the lower but not the higher end of the Hb, regardless of the time point and methods Hb concentrations been measured (Kimberly et al., 2013). It was widely accepted that women often had worse outcomes after suffering a stroke compared to men. A study focusing on the relationship between Hb and clinical outcome measured by mRS found that sex differences in stroke outcome were linked to lower Hb level, which was more prevalent in women (Park et al., 2013).

However, more detailed information on the expression levels of Hb related genes was still lacking which might be caused by the fact that Hb was in mature red blood cells with no nucleus. In other words, the concentration of Hb was determined by the genes’ expression several hours ago which may make the research more difficult to perform. However, this time gap between gene expressions and protein concentration could make these genes as predictive bio-markers for IS outcome. Compared with commonly used clinical scales such as NIH Stroke Scale (NIHSS) score, modified Rankin Scale (mRS), and biochemical tests such as routine blood tests that could describe the current recovery degree of patients, the bio-markers on gene levels could act as an early predictor for the IS patients which may help reduce the chance or severity of disability and chance of recurrence.

In this study, we chose 28 IS patients, of which 12 were suffering from anemia. Statistical analysis results showed that the outcome of the patients was different when dividing them into two groups characterized by Hb concentration. 2 sex and age matched patients were first chosen to perform RNA-seq analyses twice on different time points, after which the Hb counts were tested at least 24 h after sequencing. Results showed that the outcome of anemia patients was poor compared with the other patients. Then two other patients were chosen to exclude other factors besides Hbs. The results showed that the low value of Hb under 13 g/dL in men was closely related to the poor outcome of IS patients. Different expressed Hb related genes were tested and six genes were shown to be positively correlated with the recovery degree of IS patients: ELANE, FGF23, HBB, PIEZO1, RASA4, and PRTN3. Gene CPM was shown to be correlated with clinical outcome negatively. All of the seven genes were validated to be related to stroke using real-time PCR or literature search. Taken these together, these genes could be considered as new predictors for the recovery of IS patients.

## Materials and Methods

**FIGURE 1:**
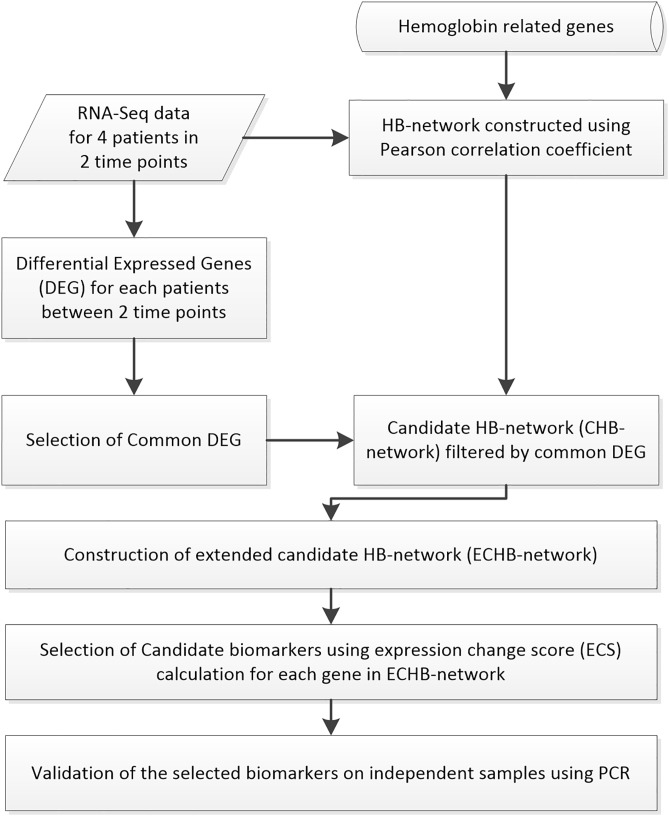
Framework of this work.

The framework of this work could be divided into several steps shown in Figure 1, which will be elaborated on in the remainder of this section.

### Clinical Samples

Whole blood samples were collected from 46 IS patients under different stages to perform analyses on three levels as follows:

(1) Clinical information analyses group (C-group): all the 28 IS patients in this group were in non-acute period (at least 10 days after the first onset occurred). They were divided into two sub-groups based on whether they were diagnosis as anemia according to the World Health Organization (WHO) criteria (Hb concentration < 13 g/dL in men and < 12 g/dl in women). The anemia ischemic stroke group (ISA) was composed of 12 patients while the non-anemia ischemic stroke group (non-ISA) contained 16 patients.
(2) RNA-Seq data analyses group (R-group): four patients in C-group were chosen including 3 non-ISA patients (marked as P1, P3, and P4) and 1 ISA patient (marked as P2). The basic clinical characters (including age, time intervals, and medical history) of P1 and P2 were similar. P3 and P4 were chosen to exclude the influence of initial condition (mRs, NIHSS, and myodynamia) when admitted.
(3) PCR analyses group (P-group): nine IS patients whose first onset occurred in May, 2018 and 9 control were chosen to perform real-time PCR for the validation of selected bio-markers. The patients were divided into two groups: 3 in ISA, 6 in non-ISA. 5 TIA patients and 4 healthy people were divided into control group.

### Data Generation

Besides Hb concentration, different types of data sets were generated from the three clinical sample groups as follows:

(1) C-group: the NIH Stroke Scale (NIHSS) score, pre-stroke disability, and clinical outcome defined using the modified Rankin Scale (mRS), BI (Barthel Index), and Myodynamia (including four levels) of all the 28 patients were collected (see Supplementary Table S1 for details). The higher scores of NIHSS, mRS or Myodynamia indicated the worse outcome of IS patients while a lower score of BI indicated a worse outcome.
(2) R-group: Illumina Hiseq2500 V4 was used to perform the RNAseq analyses on the whole blood samples for each patients on two different time points. The raw sequence data reported in this paper has been deposited in the Genome Sequence Archive (Wang et al., 2017b) in Big Data Center Members (2018), Beijing Institute of Genomics (BIG), Chinese Academy of Sciences, with accession number CRA001420 and is publicly accessible at http://bigd.big.ac.cn/gsa, as well as in the NCBI SRA repository with accession number PRJNA533989. The HB counts were tested and recorded at least 24 h after sequencing.
(3) P-group: the total RNA was extracted from blood samples using TRIzol LS according to the manufacturer’s protocol. The purified RNA was conveyed to cDNA with Golden 1st cDNA Synthesis Kit (with gDNA Remover). A SYBR Green fluorescent quantitative PCR Kit was used for real-time PCR analysis. The amplification program used in reactions were: 95°C for 15 min, 95°C for 10 s, 40 cycles of 30 s 60°C annealing.

The study was approved by SIAT Institutional Review Committee with IRB number SIAT-IRB-16515-H0107 and all the procedures were in accordance with the SIAT-IRB guidelines and the Declaration of Helsinki.

### Statistical Analyses for C-Group

The baseline characteristics of the patients were compared between the ISA and non-ISA groups using the Student’s *t*- test for continuous variables and the Pearson χ^2^ tests for categorical variables. The categorical variables including sex and medical history for each group of patients were reported as percentages, and continuous variables were reported as mean ± SD.

Regression analyses were performed using linear models to explore the relationships between the Hb count and NIHSS, mRS, BI, and Myodynamia. A small Pr( > | t| ) indicated a significant correlation between Hb counts and the corresponding factors mentioned above.

### Bioinformatics Analyses for R-Group

(1) Differential expression analyses: the sequencing raw datasets generated in R-group were analyzed using Tophat2 and Cufflinks. The differential expressed genes (DEG) between the two different time points for a same patient were identified using Cuffdiff with *q*-value not over 0.05. The biological DEG changes were further analyzed using Fold Change (FC) methods, which was the ratio between two time points based on the calculation of the average expression values. The common DEGs were selected as initial candidate bio-markers.

(2) Hemoglobin pattern analyses: the expression of 141 hemoglobin related genes retrieved by using ‘hemoglobin’ as key word in NCBI Gene with at least one expression over 0 in the 8 samples we collected were selected. A co-expression network was constructed by calculating the Pearson correlation coefficient for each pair of the 141 genes. All the pairs with *p*-value not over 0.05 were kept to construct the HB-network (hemoglobin network).

(3) Candidate HB-network (CHB-network) construction: the common DEGs were mapped into the HB-network. All the genes connecting to the common DEGs in the HB-network were kept to form the CHB-network.

(4) Extended CHB-network (ECHB-network) construction and analyses: all the genes in the CHB-network were mapped into the HB-network. Any genes connecting to any of them were kept to construct the ECHB-network. The topological analyses were performed using a R package “iGraph.” Each gene in the ECHB-network were considered as candidate bio-markers, an expression changed score (ECS) were calculated as follows:

$$\text{ECS}=(E_{2}-E_{1})/E_{1}$$

Of which, *E*_2_ presented the expression value of the gene on time-point 2 while *E*_1_ presented the expression value of the gene on time-point 1. This score reflected the changes each genes occurs between the two time points that could help estimate the biological roles of different genes. DAVID was used to perform the functional analyses for these bio-markers.

### Real-Time PCR Analysis for P-Group

The relative differences in expression between the four different groups were measured using ΔΔcycle time (CT) values: the CT values of the interested genes were semi-quantitative corrected using the internal reference gene β-actin. The expression of each gene was reported as mean ± SD. The Wilcoxon rank test was used to perform the statistical analysis.

## Results and Discussion

### Baseline Characteristics of Patients in C-Group

The baseline characteristics of the 28 patients in C-group were listed in Table 1. There were no significant differences (with *p*-value over 0.05) between the two groups on the characteristics of sex, lateral upper limb-distal, hypertension, diabetes mellitus, hyperlipemia, hyperuricemia, cerebral arteriosclerosis, atrial fibrillation, coronary disease, and carotid atherosclerosis. The regression analysis between Hb count and these baseline characters showed similar results: there were no significant associations between Hb counts and sex, Lateral upper limb-proximal, Lateral upper limb-distal, and all the Medical history besides pulmonary infection. It was interesting that the relationships between stroke, Hb counts, and pulmonary infection were different from other related diseases which might be explained by a ‘double hit model’ showing reasons of stroke-induced respiratory distress syndrome (Mascia, 2009).

**Table 1:** Baseline characteristics.

Characteristics	Non-anemia (*n* = 16)	Anemia (*n* = 12)	*p*-value	Pr( > | t| )
Sex			0.9470	0.858
Female	4 (25%)	2 (16.67%)		
Male	12 (75%)	10 (83.33%)		
Age	60.25 ± 11.1325	70.1667 ± 9.0135	0.0151^∗^	0.0863
NIHSS	7.75 ± 3.8730	12.6667 ± 2.6054	0.0005^∗∗∗^	0.00081^∗∗∗^
mRs	3.5625 ± 0.6292	4.5833 ± 0.5149	7.25E-05^∗∗∗∗^	0.000236^∗∗∗^
BI	55.3125 ± 21.5614	19.5833 ± 14.6874	1.97E-05^∗∗∗∗^	0.000155^∗∗∗^
**Myodynamia**				
Lateral upper limb-proximal	2.3934 ± 1.9767	0.8333 ± 0.9614	0.0114^∗^	0.162
Lateral upper limb-distal	2.2125 ± 1.9544	1.125 ± 1.5393	0.1117	0.17
Lateral lower limb-proximal	3.2322 ± 1.6744	1.775 ± 1.6864	0.0327^∗^	0.0545
Lateral lower limb- distal	3.10625 ± 1.8024	1.6917 ± 1.7516	0.0474^∗^	0.0686
**Medical history**				
Hypertension	12 (75%)	7 (58.33%)	0.5991	0.751
Diabetes mellitus	3 (18.75%)	4 (33.33%)	0.6592	0.654
Hyperlipemia	3 (18.75%)	2 (16.67%)	1	0.802
Hyperuricemia	0 (0.00%)	1 (8.33%)	0.8831	0.673
Cerebral arteriosclerosis	1 (6.25%)	4 (33.33%)	0.1760	0.162
Atrial fibrillation	2 (12.5%)	2 (16.67%)	1	0.997
Pulmonary infection	2 (12.5%)	9 (75%)	0.00311^∗∗^	0.0223^∗^
Coronary disease	2 (12.5%)	1 (8.33%)	1	0.335
Carotid atherosclerosis	2 (12.5%)	4 (33.33%)	0.38715	0.527

*Significance codes: ^∗^ for p-values less than 0.05, ^∗∗^ for p-values less than 0.01, ^∗∗∗^ for p-values less than 0.001, and ^∗∗∗^ for p-values less than 0.0001.*

Compared with this, the patients in ISA group were older than those in non-ISA group. Importantly, the higher NIHSS, mRs, and lower BI of ISA group compared with non-ISA showing the worse outcome of ISA patients. Besides, the myodynamia scores of the ISA group were lower than the non-ISA group. These results exhibited the important role of anemia played in the recovery of IS. The patients with lower HB counts were likely to have a worse outcome. The regression analysis results listed in Table 1 show the close relationship between Hb counts and the outcome of IS patients with smaller Pr( > | t| ) values.

### Differential Expression Analyses in R-Group

Differential expression analyses were performed to find the biomarkers of IS based on the two whole blood samples extracted from same patients on different time points in R-group. Patients P1 in non-ISA and P2 in ISA were chosen for this purpose since their basic clinical characters were similar as follows:

(1) Age: both under 65 years old. Besides age-matched, this basic feature could exclude the results caused by aging which was shown to be an important factor for the IS outcome.
(2) Time intervals: the two blood extracting time-points of P1 were 32 and 56 days after the first onset of IS while for P2 were 34 and 54 days, correspondingly. The similar time intervals of the two patients could exclude the influence caused by different stages of IS which had been shown to be of great importance in the IS outcome (Wang et al., 2017a).
(3) Medical history: both P1 and P2 suffered from hypertension which was considered as an independent risk factor for the worse outcome of IS. However, the outcomes of the two patients were different: P1 was shown to have better outcome with smaller mRs, higher BI and myodynamia compared with P2 (Table 2). The minimum HB counts of P1 and P2 were different on both of the two time points which indicated us that this could a possible reason for the different outcomes.

**Table 2:** Patients information.

Patient ID	P1-61	P2-64	P3-37	P4-60
Age	51	62	71	86
NIHSS (admitted)	2	14	9	10
mRs (discharged)	2	5	4	4
BI (discharged)	95	15	75	40
**Myodynamia (discharged)**				
Lateral upper limb-proximal	5	1	3.5	1
Lateral upper limb-distal	5	0	1	1
Lateral lower limb-proximal	4.8	0	4	1
Lateral lower limb- distal	4.8	0	4	1
**Time interval (days)**				
Between the first onset of IS and the first blood taken	32	34	48	58
Between the first onset of IS and the second blood taken	56	54	77	83
**Minimum HB count**				
Before the first blood taken date	156	73	142	142
Before the second blood taken date	133	60	148	136
Differential expressed genes between the two time points	0	8	48	30

Despite the above correlation, we still cannot make the conclusion that different outcomes were related with HB counts since the initial conditions of P1 and P2 were different: the NIHSS of P1 was 2 while P2 was 5 showing that the condition of P1 was much better than P2 when admitted. To exclude this influence, we chose two other patients (marked as P3 and P4) from the non-ISA group with higher NIHSS scores (9 and 10) respectively. Interestingly, we found similar results as follows:

(1) P3 was shown to have better outcome than P2 with lower mRs, higher BI and myodynamia when discharged. The minimum HB count of P3 was 142, higher than P2.
(2) P4 was similar to P2 on myodynamia evaluations. However, the smaller mRs and higher BI also showed better outcome of P4 than P2. Similarly as P1, P3, the minimum HB count of P4 was 136, higher than P2. The outcome of P4 may be a result of the patient’s age, as this patient was 86 years old, commonly considered by most to be very old.

Under the threshold of *q*-value ≤ 0.05, the numbers of DEGs were small: 0 for P1, 8 for P2, 48 for P3, and 30 for P4 between the two time points. The common DEGs between P2 and P3 were PRKCSH, HBD, and HBB. The common DEGs between P2 and P4 were DHPS, CFD, IRF9, FTH1P10, and HBB. HBB was the only common DEGs among P2, P3, and P4. The fold change calculating for HBB showed that for better outcome of P1 and P3, the fold change value was positive (2.42268 for P1 and 4.59503 for P3). Compared with this, the fold change value for worse outcomes was negative (−3.43035 for P2 and −3.35511 for P4). This indicated us that the decrease on HBB expression levels were closely related to the worse outcome of IS, especially in anemia or older IS patients.

### Correlations Between Candidate Bio-Markers and IS Outcome

**FIGURE 2:**
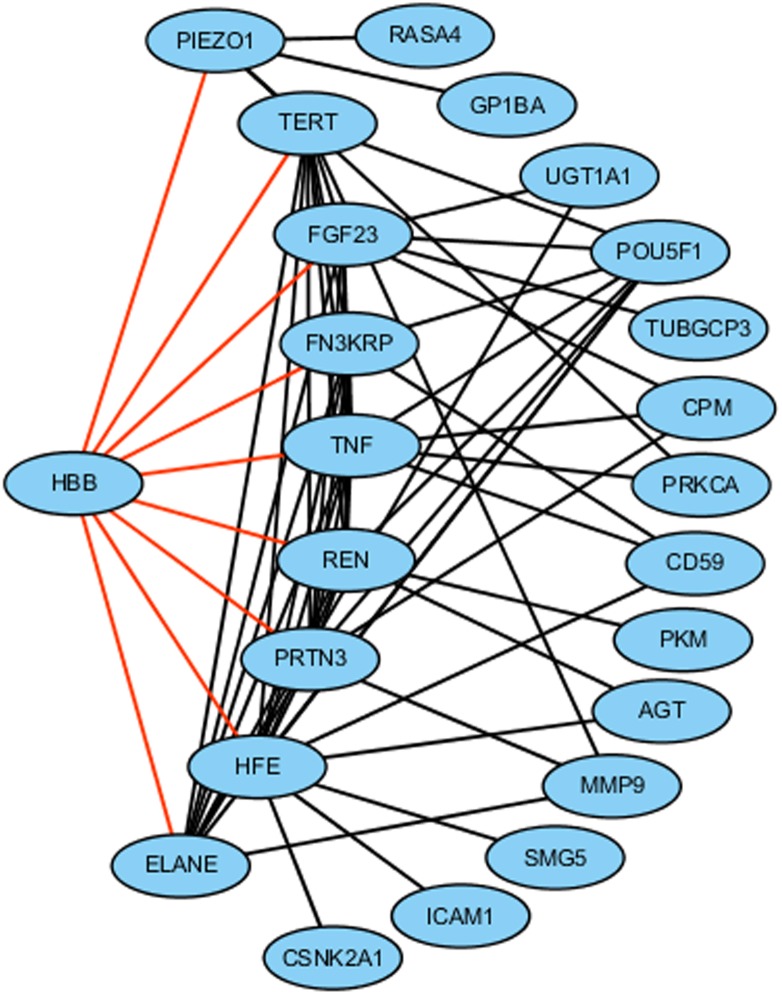
Network composed of 23 Hb related genes. Nine genes directly connected with HBB were shown by the black border lines. Different shapes represent the relationships between these genes and IS as follows: HBB-diamond. To be validated-rectangle; stroke biomarker-ellipse, stroke-related risk factor-octagon.

The HB-network was composed of 141 nodes (hemoglobin related genes) and 977 edges between them. HBB was found to be connected to nine genes as follows: PIEZO1, FGF23, ELANE, PRTN3, FN3KRP, TERT, HFE, TNF, and REN (see Figure 2 for details). These 10 genes formed the CHB-network. 6 of these genes were shown to be related to stroke as follows: (1) PIEZO1 was involved in hypertension-dependent arterial re-modeling (Retailleau et al., 2015). (2) FGF23 was a risk factor for overall stroke (Wright et al., 2014). (3) TERT was a determinant of risk of IS in the Atherosclerosis Risk in Communities (ARIC) study (Bressler et al., 2015). (4) HFE may play a role in modifying the relationship between smoking and stroke (Njajou et al., 2002). (5) TNF was differently expressed between stroke and controls (Bokhari et al., 2014). (6) REN was an important part in the renin angiotensin aldosterone system (RAAS), which played important roles in acute IS. Besides, the change of RAAS was shown to be related to the increased blood pressure (Back et al., 2015).

We further extended the CHB-network by finding out the neighbor of the 9 nodes and got other 14 nodes: AGT, CD59, CPM, ICAM1, MMP9, POU5F1, PRKCA, SMG5, UGT1A1, CSNK2A1, RASA4, GP1BA, TUBGCP3, and PKM (see Figure 2 for details). These 24 genes formed the ECHB-network. Their functional analysis results based on KEGG pathway using DAVID were shown in Supplementary Table S2. seven genes were shown to be related to stroke as follows: (1) AGT: its polymorphisms were proven to be related to the risk of IS in a meta-analysis in the Chinese population (Gao et al., 2015). (2) CD59: 50% patients with three additional mutations in CD59 were shown to have recurrent strokes (Tabib et al., 2017). (3) ICAM1: a neuro-inflammatory biomarker in post-stroke (Deddens et al., 2013). (4) MMP9: the combination therapies of MMP-9 inhibitor along with tPA was proven to be beneficial in IS (Chaturvedi and Kaczmarek, 2014). (5) PRKCA was related to the blood pressure (Shahin and Johnson, 2016). (6) RASA4 was involved in Ras signaling pathway which contributed to neuro-protective signaling cascades in stroke (Shi et al., 2011). (7) GP1BA was considered as one of the candidate ‘stroke risk’ genes affecting hemostasis (Stankovic and Majkic-Singh, 2010).

As shown in Table 2, the patients P1 and P3 had better outcomes compared with P2 and P4. We than compared the ranking of these genes based on their ECSs to find the correlations between them and the clinical outcome. As shown in Table 3, six genes were shown to be positively correlated with the recovery degree of IS patients: ELANE, FGF23, HBB, PIEZO1, RASA4, and PRTN3. Gene CPM was shown to be correlated with clinical outcome negatively. Of which, the relationships between stroke and PIEZO1, FGF23, RASA4 had been validated in former researches as mentioned above (Shi et al., 2011; Wright et al., 2014; Retailleau et al., 2015).

**Table 3:** Expression changed score and correlations with IS outcome for seven hemoglobin related genes.

Gene symbol	P1-61	P2-64	P3-37	P4-60	Correlation
ELANE	0.583441373	−0.876154889	36.11814293	−0.71205701	Positive
FGF23	0.195748708	−0.068076233	1.585274991	−0.384025361	Positive
HBB	5.019638524	−0.903588029	20.79802103	−0.902093625	Positive
PIEZO1	0.132460873	−0.130037547	0.45536621	−0.478729345	Positive
RASA4	3.743960021	−0.0797773	1.743071554	−1	Positive
PRTN3	0.285708006	−0.89533385	26.2326035	−0.767875709	Positive
CPM	−0.366842748	0.196248494	−0.308800687	−0.032850712	Negative

### Biological Validation of Candidate Bio-Markers

**FIGURE 3:**
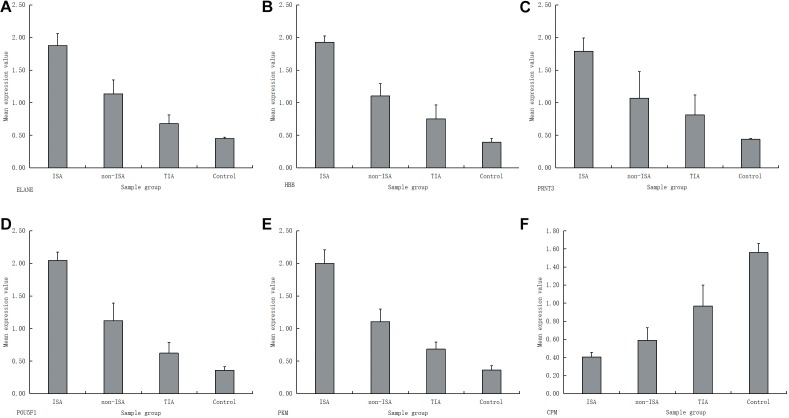
RT-PCR results for six genes with significant differences between different sample groups; **(A)** ELANE; **(B)** HBB; **(C)** PRTN3; **(D)** POU5F1; **(E)** PKM; **(F)** CPM.

Real-time PCR was performed for the following genes, which were identified as candidate bio-markers without supporting literature: ELANE, HBB, PRTN3, CPM, FN3KRP, POU5F1, SMG5, UGT1A1, CSNK2A1, TUBGCP3, and PKM. Of which, ELANE, HBB, PRTN3, POU5F1, and PKM were shown to be up-regulated in IS patients in both ISA and non-ISA groups compared with the control group. It was interesting to find out that the rank of the genes’ mean expression values were: ISA, non-ISA, TIA, control (see Figure 3A–E for details). Compared with this, CPM was shown to have trend to the contray with the mean expression rank as: control, TIA, non-ISA, ISA (see Figure 3F), which was in accordance with our results listed in Table 3. Taken together, we believe that these genes could be considered as new predictors for the recovery of IS patients.

## Ethics Statement

The study was approved by SIAT Institutional Review Committee with IRB number SIAT-IRB-16515-H0107 and all the procedures were in accordance with the SIAT-IRB guidelines and the Declaration of Helsinki.

## Author Contributions

YW performed the bio-informatics analyses and wrote the manuscript. XH collected all the blood samples for sequencing. JL analyzed the clinical information. XZ performed the PCR experiment. HY directed the clinical analyses and revised the manuscript. YC directed the bio-informatics analyses and revised the manuscript. All authors agreed to be accountable for the content of the work.

## Conflict of Interest Statement

The authors declare that the research was conducted in the absence of any commercial or financial relationships that could be construed as a potential conflict of interest.

## Supplementary Material

TABLE S1 Clinical information of patients.

TABLE S2 Common pathways of different groups.
